# Supplementary material for: Using Random Effect Models to Produce Robust Estimates of Death Rates in COVID-19 Data
Source: Int J Environ Res Public Health. 2022 Nov 14;19(22):14960. doi: 10.3390/ijerph192214960 (PMC9690214; doi:10.3390/ijerph192214960)
Supplement: Supplementary file 1 [file ijerph-19-14960-s001.zip › supp-S1.pdf]

Supplementary material for:

Using random effect models to produce robust estimates of death rates in Covid-19 data

A. Almohaimeed, J. Einbeck, N. Qarmalah, H. Alkhidir

Section S1: Supplementary output tables

Table S1: Raw and fitted cases, case rates, deaths, and death rates, on Tuesday, 21 June 2022 (Part 1)

location	population	cases	fitted cases	raw case rate	fitted case rate	deaths	fitted deaths	raw death rate	fitted death rate
Afghanistan	40099462	83	91.839	0.0000021	0.0000023	1	0.359	0.0120482	0.0039064
Albania	2854710	219	229.240	0.0000767	0.0000803	0	0.251	0.0000000	0.0010937
Algeria	44177969	8	2.766	0.0000002	0.0000001	0	0.005	0.0000000	0.0019397
Andorra	79034	0	0.013	0.0000000	0.0000002	0	0.000	NaN	0.0019828
Angola	34503774	0	2.160	0.0000000	0.0000001	0	0.004	NaN	0.0019489
Anguilla	15753	0	0.005	0.0000000	0.0000003	0	0.000	NaN	0.0019829
Antigua and Barbuda	93220	0	0.014	0.0000000	0.0000002	0	0.000	NaN	0.0019828
Argentina	45276780	0	2.835	0.0000000	0.0000001	0	0.005	NaN	0.0019386
Armenia	2790974	0	0.183	0.0000000	0.0000001	0	0.000	NaN	0.0019801
Aruba	106536	0	0.016	0.0000000	0.0000001	0	0.000	NaN	0.0019828
Australia	25921089	32895	36211.746	0.0012690	0.0013970	62	75.118	0.0018848	0.0020744
Austria	8922082	5233	5305.067	0.0005865	0.0005946	5	4.154	0.0009555	0.0007830
Azerbaijan	10312992	21	23.619	0.0000020	0.0000023	0	0.040	0.0000000	0.0016807
Bahamas	407906	34	33.705	0.0000834	0.0000826	0	0.054	0.0000000	0.0015891
Bahrain	1463265	2078	2044.180	0.0014201	0.0013970	0	1.553	0.0000000	0.0007595
Bangladesh	169356251	874	973.659	0.0000052	0.0000057	2	1.425	0.0022883	0.0014636
Barbados	281200	145	146.826	0.0005156	0.0005221	0	0.173	0.0000000	0.0011807
Belarus	9578168	0	0.600	0.0000000	0.0000001	0	0.001	NaN	0.0019734
Belgium	11611420	0	0.727	0.0000000	0.0000001	0	0.001	NaN	0.0019714
Belize	400031	277	238.169	0.0006924	0.0005954	1	0.433	0.0036101	0.0018169
Benin	12996895	0	0.814	0.0000000	0.0000001	0	0.002	NaN	0.0019700
Bermuda	64185	0	0.011	0.0000000	0.0000002	0	0.000	NaN	0.0019828
Bhutan	777486	0	0.064	0.0000000	0.0000001	0	0.000	NaN	0.0019820
Bolivia	12079472	0	0.756	0.0000000	0.0000001	0	0.001	NaN	0.0019709
Bonaire Sint Eustatius and Saba	26706	39	38.272	0.0014603	0.0014331	0	0.059	0.0000000	0.0015532
Bosnia and Herzegovina	3270943	6	6.462	0.0000018	0.0000020	0	0.012	0.0000000	0.0018854
Botswana	2588423	4286	4501.719	0.0016558	0.0017392	10	9.488	0.0023332	0.0021077
Brazil	214326223	64362	63938.974	0.0003003	0.0002983	229	263.090	0.0035580	0.0041147
British Virgin Islands	31122	0	0.007	0.0000000	0.0000002	0	0.000	NaN	0.0019829
Brunei	445373	752	774.580	0.0016885	0.0017392	0	0.673	0.0000000	0.0008686
Bulgaria	6885868	310	313.374	0.0000450	0.0000455	3	1.771	0.0096774	0.0056519
Burkina Faso	22100683	0	1.384	0.0000000	0.0000001	0	0.003	NaN	0.0019610
Burundi	12551213	0	0.786	0.0000000	0.0000001	0	0.002	NaN	0.0019704
Cambodia	16589023	0	1.039	0.0000000	0.0000001	0	0.002	NaN	0.0019664
Cameroon	27198628	0	1.703	0.0000000	0.0000001	0	0.003	NaN	0.0019560
Canada	38155012	2430	2334.981	0.0000637	0.0000612	10	8.746	0.0041152	0.0037454

Table S2: Raw and fitted cases, case rates, deaths, and death rates, on Tuesday, 21 June 2022 (Part 2)

location	population	cases	fitted cases	raw case rate	fitted case rate	deaths	fitted deaths	raw death rate	fitted death rate
Cape Verde	587925	196	191.477	0.0003334	0.0003257	0	0.216	0.0000000	0.0011273
Cayman Islands	68136	0	0.012	0.0000000	0.0000002	0	0.000	NaN	0.0019828
Central African Republic	5457154	0	0.344	0.0000000	0.0000001	0	0.001	NaN	0.0019775
Chad	17179740	0	1.076	0.0000000	0.0000001	0	0.002	NaN	0.0019659
Chile	19493184	4892	4789.462	0.0002510	0.0002457	9	8.904	0.0018397	0.0018591
China	1425893464	133	89.278	0.0000001	0.0000001	0	0.116	0.0000000	0.0013028
Colombia	51516562	0	3.226	0.0000000	0.0000001	0	0.006	NaN	0.0019327
Comoros	821626	0	0.066	0.0000000	0.0000001	0	0.000	NaN	0.0019819
Congo	5835806	0	0.367	0.0000000	0.0000001	0	0.001	NaN	0.0019771
Cook Islands	17003	4	3.295	0.0002353	0.0001938	0	0.006	0.0000000	0.0019316
Costa Rica	5153957	0	0.326	0.0000000	0.0000001	0	0.001	NaN	0.0019778
Cote d'Ivoire	27478249	62	62.933	0.0000023	0.0000023	0	0.088	0.0000000	0.0014044
Croatia	4060135	397	350.778	0.0000978	0.0000864	4	3.491	0.0100756	0.0099511
Cuba	11256372	17	21.549	0.0000015	0.0000019	0	0.037	0.0000000	0.0017018
Curacao	190338	0	0.024	0.0000000	0.0000001	0	0.000	NaN	0.0019826
Cyprus	896007	0	0.071	0.0000000	0.0000001	0	0.000	NaN	0.0019819
Czechia	10510750	609	643.229	0.0000579	0.0000612	0	0.580	0.0000000	0.0009010
Democratic Republic of Congo	95894118	0	6.004	0.0000000	0.0000001	0	0.011	NaN	0.0018919
Denmark	5854240	2141	1944.376	0.0003657	0.0003321	3	2.301	0.0014012	0.0011833
Djibouti	1105557	0	0.083	0.0000000	0.0000001	0	0.000	NaN	0.0019817
Dominica	72412	0	0.012	0.0000000	0.0000002	0	0.000	NaN	0.0019828
Dominican Republic	11117874	515	505.971	0.0000463	0.0000455	0	0.479	0.0000000	0.0009457
Ecuador	17797737	2184	2068.915	0.0001227	0.0001162	2	1.839	0.0009158	0.0008889
Egypt	109262178	0	6.841	0.0000000	0.0000001	0	0.013	NaN	0.0018801
El Salvador	6314167	5512	5529.857	0.0008730	0.0008758	1	4.091	0.0001814	0.0007398
Equatorial Guinea	1634466	4	3.742	0.0000024	0.0000023	0	0.007	0.0000000	0.0019250
Eritrea	3620312	5	4.876	0.0000014	0.0000013	0	0.009	0.0000000	0.0019082
Estonia	1328701	822	790.045	0.0006186	0.0005946	2	1.339	0.0024331	0.0016953
Eswatini	1192271	15	13.331	0.0000126	0.0000112	0	0.024	0.0000000	0.0017949
Ethiopia	120283026	511	477.493	0.0000042	0.0000040	1	0.661	0.0019569	0.0013847
Faeroe Islands	52888	0	0.010	0.0000000	0.0000002	0	0.000	NaN	0.0019829
Falkland Islands	3764	0	0.002	0.0000000	0.0000006	0	0.000	NaN	0.0019830
Fiji	924610	0	0.073	0.0000000	0.0000001	0	0.000	NaN	0.0019818
Finland	5535992	0	0.349	0.0000000	0.0000001	0	0.001	NaN	0.0019774
France	67422000	95317	94188.494	0.0014137	0.0013970	56	69.626	0.0005875	0.0007392
French Polynesia	304032	0	0.033	0.0000000	0.0000001	0	0.000	NaN	0.0019825

Table S3: Raw and fitted cases, case rates, deaths, and death rates, on Tuesday, 21 June 2022 (Part 3)

location	population	cases	fitted cases	raw case rate	fitted case rate	deaths	fitted deaths	raw death rate	fitted death rate
Gabon	2341179	0	0.157	0.0000000	0.0000001	0	0.000	NaN	0.0019805
Gambia	2639916	0	0.174	0.0000000	0.0000001	0	0.000	NaN	0.0019802
Georgia	3757980	0	0.241	0.0000000	0.0000001	0	0.000	NaN	0.0019791
Germany	83408554	119232	116521.701	0.0014295	0.0013970	104	86.136	0.0008722	0.0007392
Ghana	32833031	0	2.056	0.0000000	0.0000001	0	0.004	NaN	0.0019506
Gibraltar	32670	0	0.007	0.0000000	0.0000002	0	0.000	NaN	0.0019829
Greece	10445365	12580	11305.947	0.0012044	0.0010824	16	15.919	0.0012719	0.0014080
Greenland	56243	0	0.010	0.0000000	0.0000002	0	0.000	NaN	0.0019828
Grenada	124610	90	84.579	0.0007223	0.0006787	1	0.356	0.0111111	0.0042149
Guatemala	17608483	0	1.103	0.0000000	0.0000001	0	0.002	NaN	0.0019654
Guernsey	63065	0	0.011	0.0000000	0.0000002	0	0.000	NaN	0.0019828
Guinea	13531906	0	0.847	0.0000000	0.0000001	0	0.002	NaN	0.0019695
Guinea-Bissau	2060721	40	37.998	0.0000194	0.0000184	0	0.059	0.0000000	0.0015553
Guyana	804567	82	80.624	0.0001019	0.0001002	0	0.107	0.0000000	0.0013314
Haiti	11447569	0	0.717	0.0000000	0.0000001	0	0.001	NaN	0.0019715
Honduras	10278346	0	0.644	0.0000000	0.0000001	0	0.001	NaN	0.0019727
Hong Kong	7494578	1198	1202.386	0.0001598	0.0001604	0	0.968	0.0000000	0.0008054
Hungary	9709786	0	0.608	0.0000000	0.0000001	0	0.001	NaN	0.0019733
Iceland	370335	952	888.410	0.0025706	0.0023989	0	0.752	0.0000000	0.0008467
India	1407563842	12249	12125.725	0.0000087	0.0000086	13	9.194	0.0010613	0.0007582
Indonesia	273753191	1678	1573.856	0.0000061	0.0000057	5	4.240	0.0029797	0.0026941
Iran	87923432	187	201.368	0.0000021	0.0000023	4	4.060	0.0213904	0.0201631
Iraq	43533592	751	745.712	0.0000173	0.0000171	0	0.653	0.0000000	0.0008750
Ireland	4986526	0	0.315	0.0000000	0.0000001	0	0.001	NaN	0.0019779
Isle of Man	84263	0	0.013	0.0000000	0.0000002	0	0.000	NaN	0.0019828
Israel	9291000	10669	10056.475	0.0011483	0.0010824	0	7.434	0.0000000	0.0007392
Italy	59240330	63264	64121.075	0.0010679	0.0010824	62	47.400	0.0009800	0.0007392
Jamaica	2827694	61	59.796	0.0000216	0.0000211	0	0.085	0.0000000	0.0014199
Japan	124612530	15367	14485.700	0.0001233	0.0001162	17	11.424	0.0011063	0.0007886
Jersey	109618	0	0.016	0.0000000	0.0000001	0	0.000	NaN	0.0019828
Jordan	11148278	0	0.698	0.0000000	0.0000001	0	0.001	NaN	0.0019718
Kazakhstan	19196465	0	1.202	0.0000000	0.0000001	0	0.002	NaN	0.0019639
Kenya	53005614	419	456.627	0.0000079	0.0000086	0	0.441	0.0000000	0.0009653
Kiribati	128874	0	0.018	0.0000000	0.0000001	0	0.000	NaN	0.0019827
Kosovo	1782115	13	12.914	0.0000073	0.0000072	0	0.023	0.0000000	0.0018000
Kuwait	4250114	0	0.271	0.0000000	0.0000001	0	0.001	NaN	0.0019787

Table S4: Raw and fitted cases, case rates, deaths, and death rates, on Tuesday, 21 June 2022 (Part 4)

location	population	cases	fitted cases	raw case rate	fitted case rate	deaths	fitted deaths	raw death rate	fitted death rate
Kyrgyzstan	6527743	0	0.410	0.0000000	0.0000001	0	0.001	NaN	0.0019764
Laos	7425058	6	5.667	0.0000008	0.0000008	0	0.011	0.0000000	0.0018968
Latvia	1873919	317	316.707	0.0001692	0.0001690	5	6.189	0.0157729	0.0195408
Lebanon	5592631	557	519.251	0.0000996	0.0000928	4	2.021	0.0071813	0.0038931
Lesotho	2281454	0	0.153	0.0000000	0.0000001	0	0.000	NaN	0.0019805
Liberia	5193416	0	0.328	0.0000000	0.0000001	0	0.001	NaN	0.0019777
Libya	6735277	0	0.423	0.0000000	0.0000001	0	0.001	NaN	0.0019762
Liechtenstein	39039	32	33.424	0.0008197	0.0008562	0	0.053	0.0000000	0.0015915
Lithuania	2786651	397	445.206	0.0001425	0.0001598	0	0.432	0.0000000	0.0009701
Luxembourg	639321	1209	1111.891	0.0018911	0.0017392	2	1.474	0.0016543	0.0013259
Macao	686607	5	4.349	0.0000073	0.0000063	0	0.008	0.0000000	0.0019159
Madagascar	28915653	0	1.810	0.0000000	0.0000001	0	0.004	NaN	0.0019544
Malawi	19889742	24	15.393	0.0000012	0.0000008	0	0.027	0.0000000	0.0017701
Malaysia	33573874	1921	2054.628	0.0000572	0.0000612	2	1.832	0.0010411	0.0008918
Maldives	521458	0	0.048	0.0000000	0.0000001	0	0.000	NaN	0.0019822
Mali	21904983	2	1.372	0.0000001	0.0000001	0	0.003	0.0000000	0.0019612
Malta	526748	488	462.077	0.0009264	0.0008772	5	4.196	0.0102459	0.0090802
Marshall Islands	42050	0	0.008	0.0000000	0.0000002	0	0.000	NaN	0.0019829
Mauritania	4614974	14	12.159	0.0000030	0.0000026	0	0.022	0.0000000	0.0018095
Mauritius	1298915	0	0.095	0.0000000	0.0000001	1	0.001	Inf	0.0109863
Mexico	126705138	13752	14728.957	0.0001085	0.0001162	41	32.689	0.0029814	0.0022194
Micronesia (country)	113131	0	0.017	0.0000000	0.0000001	0	0.000	NaN	0.0019827
Moldova	3061506	0	0.199	0.0000000	0.0000001	0	0.000	NaN	0.0019798
Monaco	36686	56	54.522	0.0015265	0.0014862	0	0.079	0.0000000	0.0014479
Mongolia	3347782	0	0.216	0.0000000	0.0000001	0	0.000	NaN	0.0019795
Montenegro	627859	113	107.073	0.0001800	0.0001705	0	0.134	0.0000000	0.0012546
Montserrat	4417	0	0.002	0.0000000	0.0000006	0	0.000	NaN	0.0019830
Morocco	37076584	3059	3160.269	0.0000825	0.0000852	0	2.350	0.0000000	0.0007438
Mozambique	32077072	94	74.949	0.0000029	0.0000023	1	0.351	0.0106383	0.0046861
Myanmar	53798084	14	3.466	0.0000003	0.0000001	0	0.007	0.0000000	0.0019291
Namibia	2530151	0	0.168	0.0000000	0.0000001	0	0.000	NaN	0.0019803
Nauru	12512	0	0.004	0.0000000	0.0000003	0	0.000	NaN	0.0019829
Nepal	30034989	14	22.710	0.0000005	0.0000008	0	0.038	0.0000000	0.0016898
Netherlands	17501696	4793	5221.054	0.0002739	0.0002983	2	3.871	0.0004173	0.0007415
New Caledonia	287800	377	401.808	0.0013099	0.0013961	0	0.398	0.0000000	0.0009897
New Zealand	5129728	5684	5552.361	0.0011081	0.0010824	0	4.105	0.0000000	0.0007394

Table S5: Raw and fitted cases, case rates, deaths, and death rates, on Tuesday, 21 June 2022 (Part 5)

location	population	cases	fitted cases	raw case rate	fitted case rate	deaths	fitted deaths	raw death rate	fitted death rate
Nicaragua	6850540	0	0.430	0.0000000	0.0000001	0	0.001	NaN	0.0019761
Niger	25252722	0	1.581	0.0000000	0.0000001	0	0.003	NaN	0.0019579
Nigeria	213401323	138	161.359	0.0000006	0.0000008	0	0.187	0.0000000	0.0011610
North Korea	25971909	0	1.626	0.0000000	0.0000001	0	0.003	NaN	0.0019572
North Macedonia	2103330	90	95.414	0.0000428	0.0000454	0	0.123	0.0000000	0.0012847
Norway	5403021	647	628.079	0.0001197	0.0001162	0	0.569	0.0000000	0.0009053
Oman	4520471	0	0.287	0.0000000	0.0000001	0	0.001	NaN	0.0019784
Pakistan	231402116	204	174.970	0.0000009	0.0000008	0	0.200	0.0000000	0.0011447
Palau	18024	12	11.614	0.0006658	0.0006443	0	0.021	0.0000000	0.0018164
Palestine	5133392	0	0.324	0.0000000	0.0000001	0	0.001	NaN	0.0019778
Panama	4351267	2080	1978.796	0.0004780	0.0004548	4	3.220	0.0019231	0.0016273
Papua New Guinea	9949437	0	0.623	0.0000000	0.0000001	0	0.001	NaN	0.0019730
Paraguay	6703799	0	0.421	0.0000000	0.0000001	0	0.001	NaN	0.0019763
Peru	33715472	2134	2063.294	0.0000633	0.0000612	10	8.017	0.0046860	0.0038856
Philippines	113880328	407	452.076	0.0000036	0.0000040	9	9.939	0.0221130	0.0219859
Poland	38307726	374	382.532	0.0000098	0.0000100	7	8.312	0.0187166	0.0217284
Portugal	10290103	18035	17896.284	0.0017527	0.0017392	22	14.806	0.0012199	0.0008273
Qatar	2688235	662	660.498	0.0002463	0.0002457	0	0.592	0.0000000	0.0008962
Romania	19328560	0	1.210	0.0000000	0.0000001	0	0.002	NaN	0.0019637
Russia	145102755	2474	2485.548	0.0000170	0.0000171	59	54.734	0.0238480	0.0220211
Rwanda	13461888	38	31.805	0.0000028	0.0000024	0	0.051	0.0000000	0.0016050
Saint Helena	5404	0	0.003	0.0000000	0.0000005	0	0.000	NaN	0.0019830
Saint Kitts and Nevis	47606	0	0.009	0.0000000	0.0000002	0	0.000	NaN	0.0019829
Saint Lucia	179652	17	16.218	0.0000946	0.0000903	0	0.029	0.0000000	0.0017604
Saint Pierre and Miquelon	5883	0	0.003	0.0000000	0.0000005	0	0.000	NaN	0.0019830
Saint Vincent and the Grenadines	104332	39	36.521	0.0003738	0.0003500	1	0.269	0.0256410	0.0073756
Samoa	218764	275	293.934	0.0012571	0.0013436	0	0.308	0.0000000	0.0010482
San Marino	33746	68	62.682	0.0020151	0.0018575	0	0.088	0.0000000	0.0014056
Sao Tome and Principe	223107	0	0.026	0.0000000	0.0000001	0	0.000	NaN	0.0019826
Saudi Arabia	35950396	1143	1143.113	0.0000318	0.0000318	2	1.485	0.0017498	0.0012988
Senegal	16876720	1	1.057	0.0000001	0.0000001	0	0.002	0.0000000	0.0019662
Serbia	6871547	507	513.736	0.0000738	0.0000748	1	0.690	0.0019724	0.0013440
Seychelles	106470	0	0.016	0.0000000	0.0000001	0	0.000	NaN	0.0019828
Sierra Leone	8420641	0	0.528	0.0000000	0.0000001	0	0.001	NaN	0.0019746
Singapore	5453600	7109	7618.676	0.0013035	0.0013970	0	5.632	0.0000000	0.0007392
Sint Maarten (Dutch part)	44042	0	0.009	0.0000000	0.0000002	0	0.000	NaN	0.0019829
Slovakia	5447622	454	463.225	0.0000833	0.0000850	0	0.446	0.0000000	0.0009625

Table S6: Raw and fitted cases, case rates, deaths, and death rates, on Tuesday, 21 June 2022 (Part 6)

location	population	cases	fitted cases	raw case rate	fitted case rate	deaths	fitted deaths	raw death rate	fitted death rate
Slovenia	2119410	856	963.789	0.0004039	0.0004547	0	0.804	0.0000000	0.0008345
Solomon Islands	707851	0	0.059	0.0000000	0.0000001	0	0.000	NaN	0.0019821
Somalia	17065581	0	1.069	0.0000000	0.0000001	0	0.002	NaN	0.0019660
South Africa	59392255	0	3.719	0.0000000	0.0000001	20	0.082	Inf	0.0220211
South Korea	51830139	8968	9075.334	0.0001730	0.0001751	11	7.898	0.0012266	0.0008702
South Sudan	10748272	0	0.673	0.0000000	0.0000001	0	0.001	NaN	0.0019722
Spain	47486935	50235	51399.331	0.0010579	0.0010824	122	106.623	0.0024286	0.0020744
Sri Lanka	21773441	8	14.289	0.0000004	0.0000007	0	0.025	0.0000000	0.0017832
Sudan	45657202	12	3.020	0.0000003	0.0000001	0	0.006	0.0000000	0.0019358
Suriname	612984	51	50.777	0.0000832	0.0000828	4	1.112	0.0784314	0.0218901
Sweden	10467097	0	0.656	0.0000000	0.0000001	0	0.001	NaN	0.0019725
Switzerland	8691406	24720	24712.141	0.0028442	0.0028433	0	18.268	0.0000000	0.0007392
Syria	21324367	1	1.335	0.0000000	0.0000001	0	0.003	0.0000000	0.0019618
Taiwan	23859912	56366	56433.392	0.0023624	0.0023652	115	117.066	0.0020402	0.0020744
Tajikistan	9750064	0	0.611	0.0000000	0.0000001	0	0.001	NaN	0.0019732
Tanzania	63588334	0	3.981	0.0000000	0.0000001	0	0.008	NaN	0.0019214
Thailand	71601103	1714	1707.683	0.0000239	0.0000238	21	36.989	0.0122520	0.0216603
Timor	1320942	1	0.328	0.0000008	0.0000002	0	0.001	0.0000000	0.0019777
Togo	8644829	0	0.542	0.0000000	0.0000001	0	0.001	NaN	0.0019743
Tokelau	1849	0	0.002	0.0000000	0.0000009	0	0.000	NaN	0.0019830
Tonga	106017	0	0.016	0.0000000	0.0000001	0	0.000	NaN	0.0019828
Trinidad and Tobago	1525663	145	132.437	0.0000950	0.0000868	3	2.506	0.0206897	0.0189221
Tunisia	12262946	2277	2147.213	0.0001857	0.0001751	15	8.814	0.0065876	0.0041048
Turkey	84775404	0	5.308	0.0000000	0.0000001	0	0.010	NaN	0.0019019
Turks and Caicos Islands	45114	0	0.009	0.0000000	0.0000002	0	0.000	NaN	0.0019829
Uganda	45853778	42	34.671	0.0000009	0.0000008	3	0.743	0.0714286	0.0214384
Ukraine	43531422	26	32.915	0.0000006	0.0000008	1	0.253	0.0384615	0.0076954
United Arab Emirates	9365144	1556	1535.880	0.0001661	0.0001640	0	1.198	0.0000000	0.0007801
United Kingdom	67281040	16430	16530.905	0.0002442	0.0002457	105	68.020	0.0063907	0.0041147
United States	336997624	111728	111927.460	0.0003315	0.0003321	451	460.548	0.0040366	0.0041147
Uruguay	3426260	0	0.221	0.0000000	0.0000001	0	0.000	NaN	0.0019795
Uzbekistan	34081449	87	78.057	0.0000026	0.0000023	0	0.105	0.0000000	0.0013407
Vanuatu	319136	65	60.313	0.0002037	0.0001890	0	0.085	0.0000000	0.0014173
Vatican	511	0	0.001	0.0000000	0.0000016	0	0.000	NaN	0.0019830
Venezuela	28199866	127	113.631	0.0000045	0.0000040	1	0.362	0.0078740	0.0031849
Vietnam	97468028	748	839.657	0.0000077	0.0000086	0	0.718	0.0000000	0.0008555
Wallis and Futuna	11627	0	0.004	0.0000000	0.0000003	0	0.000	NaN	0.0019829
Yemen	32981641	1	2.065	0.0000000	0.0000001	0	0.004	0.0000000	0.0019504
Zambia	19473125	214	198.536	0.0000110	0.0000102	1	0.398	0.0046729	0.0020060
Zimbabwe	15993524	88	91.401	0.0000055	0.0000057	2	1.290	0.0227273	0.0141156

Table S7: Raw and fitted case and death rates, on Tuesday, 21 June 2022, relative to cases 14 days earlier (Part 1).

location	cases	fitted cases	raw case rate	fitted case rate	fitted deaths	raw death rate	fitted death rate
Afghanistan	53	65.833	0.0000013	0.0000016	0.439	0.0120482	0.0066713
Albania	53	54.046	0.0000186	0.0000189	0.070	0.0000000	0.0012918
Algeria	4	3.914	0.0000001	0.0000001	0.007	0.0000000	0.0018068
Andorra	0	0.017	0.0000000	0.0000002	0.000	NaN	0.0018613
Angola	0	3.056	0.0000000	0.0000001	0.006	NaN	0.0018185
Anguilla	0	0.007	0.0000000	0.0000004	0.000	NaN	0.0018614
Antigua and Barbuda	0	0.019	0.0000000	0.0000002	0.000	NaN	0.0018612
Argentina	0	4.010	0.0000000	0.0000001	0.007	NaN	0.0018054
Armenia	0	0.273	0.0000000	0.0000001	0.001	NaN	0.0018576
Aruba	0	0.021	0.0000000	0.0000002	0.000	NaN	0.0018612
Australia	33223	33031.390	0.0012817	0.0012743	48.139	0.0018848	0.0014574
Austria	2183	2284.134	0.0002447	0.0002560	3.953	0.0009555	0.0017305
Azerbaijan	0	0.925	0.0000000	0.0000001	0.002	0.0000000	0.0018483
Bahamas	20	19.675	0.0000490	0.0000482	0.032	0.0000000	0.0016104
Bahrain	997	1054.167	0.0006814	0.0007204	0.340	0.0000000	0.0003224
Bangladesh	54	60.609	0.0000003	0.0000004	0.722	0.0022883	0.0119116
Barbados	104	93.738	0.0003698	0.0003334	0.099	0.0000000	0.0010537
Belarus	0	0.861	0.0000000	0.0000001	0.002	NaN	0.0018492
Belgium	5944	6189.447	0.0005119	0.0005330	1.182	NaN	0.0001910
Belize	224	212.846	0.0005600	0.0005321	0.638	0.0036101	0.0029986
Benin	0	1.158	0.0000000	0.0000001	0.002	NaN	0.0018450
Bermuda	0	0.015	0.0000000	0.0000002	0.000	NaN	0.0018613
Bhutan	0	0.094	0.0000000	0.0000001	0.000	NaN	0.0018602
Bolivia	316	343.718	0.0000262	0.0000285	0.200	NaN	0.0005806
Bonaire Sint Eustatius and Saba	0	0.009	0.0000000	0.0000003	0.000	0.0000000	0.0018614
Bosnia and Herzegovina	24	28.025	0.0000073	0.0000086	0.043	0.0000000	0.0015205
Botswana	2305	2617.581	0.0008905	0.0010113	10.479	0.0023332	0.0040034
Brazil	71045	71102.062	0.0003315	0.0003317	303.065	0.0035580	0.0042624
British Virgin Islands	0	0.010	0.0000000	0.0000003	0.000	NaN	0.0018614
Brunei	380	356.248	0.0008532	0.0007999	0.203	0.0000000	0.0005711
Bulgaria	168	154.407	0.0000244	0.0000224	1.959	0.0096774	0.0126887
Burkina Faso	0	1.958	0.0000000	0.0000001	0.004	NaN	0.0018338
Burundi	0	1.119	0.0000000	0.0000001	0.002	NaN	0.0018456
Cambodia	0	1.473	0.0000000	0.0000001	0.003	NaN	0.0018406
Cameroon	0	2.409	0.0000000	0.0000001	0.004	NaN	0.0018275
Canada	2936	2917.449	0.0000769	0.0000765	10.865	0.0041152	0.0037242

Table S8: Raw and fitted case and death rates, on Tuesday, 21 June 2022, relative to cases 14 days earlier (Part 2).

location	cases	fitted.cases	raw case rate	fitted case rate	fitted deaths	raw death rate	fitted death rate
Cape Verde	45	42.797	0.0000765	0.0000728	0.059	0.0000000	0.0013820
Cayman Islands	0	0.015	0.0000000	0.0000002	0.000	NaN	0.0018613
Central African Republic	0	0.506	0.0000000	0.0000001	0.001	NaN	0.0018543
Chad	0	1.524	0.0000000	0.0000001	0.003	NaN	0.0018399
Chile	4671	4990.432	0.0002396	0.0002560	7.328	0.0018397	0.0014684
China	203	126.271	0.0000001	0.0000001	0.117	0.0000000	0.0009235
Colombia	0	4.562	0.0000000	0.0000001	0.008	NaN	0.0017979
Comoros	0	0.098	0.0000000	0.0000001	0.000	NaN	0.0018601
Congo	0	0.539	0.0000000	0.0000001	0.001	NaN	0.0018538
Cook Islands	14	13.147	0.0008234	0.0007732	0.022	0.0000000	0.0016872
Costa Rica	0	0.480	0.0000000	0.0000001	0.001	NaN	0.0018547
Cote d'Ivoire	0	2.434	0.0000000	0.0000001	0.004	0.0000000	0.0018272
Croatia	202	192.210	0.0000498	0.0000473	2.611	0.0100756	0.0135845
Cuba	13	18.382	0.0000012	0.0000016	0.030	0.0000000	0.0016251
Curacao	0	0.032	0.0000000	0.0000002	0.000	NaN	0.0018611
Cyprus	0	0.105	0.0000000	0.0000001	0.000	NaN	0.0018600
Czechia	315	319.104	0.0000300	0.0000304	0.192	0.0000000	0.0006008
Democratic Republic of Congo	0	8.492	0.0000000	0.0000001	0.015	NaN	0.0017458
Denmark	1987	1942.126	0.0003394	0.0003317	2.885	0.0014012	0.0014853
Djibouti	0	0.123	0.0000000	0.0000001	0.000	NaN	0.0018597
Dominica	0	0.016	0.0000000	0.0000002	0.000	NaN	0.0018613
Dominican Republic	1482	1574.130	0.0001333	0.0001416	0.406	0.0000000	0.0002577
Ecuador	0	1.579	0.0000000	0.0000001	0.020	0.0009158	0.0128893
Egypt	0	9.676	0.0000000	0.0000001	0.017	NaN	0.0017306
El Salvador	399	385.360	0.0000632	0.0000610	0.689	0.0001814	0.0017891
Equatorial Guinea	3	2.780	0.0000018	0.0000017	0.005	0.0000000	0.0018224
Eritrea	0	0.346	0.0000000	0.0000001	0.001	0.0000000	0.0018566
Estonia	785	708.304	0.0005908	0.0005331	1.508	0.0024331	0.0021287
Eswatini	46	44.730	0.0000386	0.0000375	0.061	0.0000000	0.0013656
Ethiopia	443	447.497	0.0000037	0.0000037	0.723	0.0019569	0.0016158
Faeroe Islands	0	0.013	0.0000000	0.0000002	0.000	NaN	0.0018613
Falkland Islands	0	0.003	0.0000000	0.0000009	0.000	NaN	0.0018615
Fiji	0	0.107	0.0000000	0.0000001	0.000	NaN	0.0018600
Finland	0	0.513	0.0000000	0.0000001	0.001	NaN	0.0018542
France	11665	11671.657	0.0001730	0.0001731	49.749	0.0005875	0.0042624
French Polynesia	0	0.046	0.0000000	0.0000002	0.000	NaN	0.0018609

Table S9: Raw and fitted case and death rates, on Tuesday, 21 June 2022, relative to cases 14 days earlier (Part 3).

location	cases	fitted.cases	raw case rate	fitted case rate	fitted deaths	raw death rate	fitted death rate
Gabon	0	0.233	0.0000000	0.0000001	0.000	NaN	0.0018582
Gambia	0	0.259	0.0000000	0.0000001	0.000	NaN	0.0018578
Georgia	511	532.030	0.0001360	0.0001416	0.250	NaN	0.0004702
Germany	84655	84348.131	0.0010149	0.0010113	122.926	0.0008722	0.0014574
Ghana	46	53.904	0.0000014	0.0000016	0.070	NaN	0.0012928
Gibraltar	0	0.010	0.0000000	0.0000003	0.000	NaN	0.0018614
Greece	5638	5568.214	0.0005398	0.0005331	17.566	0.0012719	0.0031548
Greenland	0	0.014	0.0000000	0.0000002	0.000	NaN	0.0018613
Grenada	82	83.098	0.0006581	0.0006669	0.504	0.0111111	0.0060657
Guatemala	1132	1074.583	0.0000643	0.0000610	0.343	NaN	0.0003189
Guernsey	0	0.015	0.0000000	0.0000002	0.000	NaN	0.0018613
Guinea	0	1.205	0.0000000	0.0000001	0.002	NaN	0.0018444
Guinea-Bissau	7	5.578	0.0000034	0.0000027	0.010	0.0000000	0.0017842
Guyana	128	124.596	0.0001591	0.0001549	0.116	0.0000000	0.0009292
Haiti	48	42.264	0.0000042	0.0000037	0.059	NaN	0.0013866
Honduras	0	0.922	0.0000000	0.0000001	0.002	NaN	0.0018484
Hong Kong	239	227.555	0.0000319	0.0000304	0.160	0.0000000	0.0007016
Hungary	0	0.873	0.0000000	0.0000001	0.002	NaN	0.0018491
Iceland	395	374.656	0.0010666	0.0010117	0.209	0.0000000	0.0005578
India	5233	5236.659	0.0000037	0.0000037	9.524	0.0010613	0.0018186
Indonesia	518	449.434	0.0000019	0.0000016	5.439	0.0029797	0.0121019
Iran	139	144.348	0.0000016	0.0000016	1.996	0.0213904	0.0138289
Iraq	170	161.948	0.0000039	0.0000037	0.133	0.0000000	0.0008224
Ireland	0	0.465	0.0000000	0.0000001	0.001	NaN	0.0018549
Isle of Man	0	0.018	0.0000000	0.0000002	0.000	NaN	0.0018613
Israel	5031	4952.845	0.0005415	0.0005331	0.950	0.0000000	0.0001917
Italy	28547	28548.157	0.0004819	0.0004819	41.605	0.0009800	0.0014574
Jamaica	174	172.736	0.0000615	0.0000611	0.138	0.0000000	0.0007979
Japan	17021	17643.326	0.0001366	0.0001416	25.713	0.0011063	0.0014574
Jersey	0	0.021	0.0000000	0.0000002	0.000	NaN	0.0018612
Jordan	0	0.997	0.0000000	0.0000001	0.002	NaN	0.0018473
Kazakhstan	33	31.536	0.0000017	0.0000016	0.047	NaN	0.0014853
Kenya	163	155.882	0.0000031	0.0000029	0.131	0.0000000	0.0008373
Kiribati	22	20.081	0.0001707	0.0001558	0.032	NaN	0.0016058
Kosovo	0	0.183	0.0000000	0.0000001	0.000	0.0000000	0.0018589
Kuwait	470	483.120	0.0001106	0.0001137	0.238	NaN	0.0004936

Table S10: Raw and fitted case and death rates, on Tuesday, 21 June 2022, relative to cases 14 days earlier (Part 4).

location	cases	fitted cases	raw case rate	fitted case rate	fitted deaths	raw death rate	fitted death rate
Kyrgyzstan	0	0.599	0.0000000	0.0000001	0.001	NaN	0.0018530
Laos	15	12.849	0.0000020	0.0000017	0.022	0.0000000	0.0016909
Latvia	215	213.494	0.0001147	0.0001139	2.989	0.0157729	0.0140018
Lebanon	0	0.518	0.0000000	0.0000001	0.007	0.0071813	0.0141439
Lesotho	0	0.228	0.0000000	0.0000001	0.000	NaN	0.0018583
Liberia	0	0.483	0.0000000	0.0000001	0.001	NaN	0.0018546
Libya	0	0.616	0.0000000	0.0000001	0.001	NaN	0.0018527
Liechtenstein	36	35.912	0.0009222	0.0009199	0.052	0.0000000	0.0014435
Lithuania	220	217.268	0.0000789	0.0000780	0.156	0.0000000	0.0007168
Luxembourg	0	0.081	0.0000000	0.0000001	0.001	0.0016543	0.0129086
Macao	0	0.085	0.0000000	0.0000001	0.000	0.0000000	0.0018603
Madagascar	0	2.561	0.0000000	0.0000001	0.005	NaN	0.0018254
Malawi	14	7.329	0.0000007	0.0000004	0.013	0.0000000	0.0017610
Malaysia	1128	1019.295	0.0000336	0.0000304	1.711	0.0010411	0.0016784
Maldives	0	0.069	0.0000000	0.0000001	0.000	NaN	0.0018605
Mali	4	2.224	0.0000002	0.0000001	0.004	0.0000000	0.0018301
Malta	93	87.952	0.0001766	0.0001670	1.247	0.0102459	0.0141737
Marshall Islands	0	0.011	0.0000000	0.0000003	0.000	NaN	0.0018614
Mauritania	7	7.666	0.0000015	0.0000017	0.013	0.0000000	0.0017566
Mauritius	0	0.141	0.0000000	0.0000001	0.001	Inf	0.0091016
Mexico	5110	5112.840	0.0000403	0.0000404	28.841	0.0029814	0.0056410
Micronesia (country)	0	0.022	0.0000000	0.0000002	0.000	NaN	0.0018612
Moldova	0	0.297	0.0000000	0.0000001	0.001	NaN	0.0018573
Monaco	41	40.132	0.0011176	0.0010939	0.056	0.0000000	0.0014052
Mongolia	0	0.322	0.0000000	0.0000001	0.001	NaN	0.0018569
Montenegro	71	73.841	0.0001131	0.0001176	0.086	0.0000000	0.0011595
Montserrat	0	0.004	0.0000000	0.0000008	0.000	NaN	0.0018615
Morocco	607	632.113	0.0000164	0.0000170	0.272	0.0000000	0.0004296
Mozambique	81	90.688	0.0000025	0.0000028	0.527	0.0106383	0.0058109
Myanmar	10	5.334	0.0000002	0.0000001	0.010	0.0000000	0.0017875
Namibia	0	0.250	0.0000000	0.0000001	0.000	NaN	0.0018579
Nauru	0	0.006	0.0000000	0.0000005	0.000	NaN	0.0018614
Nepal	7	4.953	0.0000002	0.0000002	0.009	0.0000000	0.0017926
Netherlands	1502	1498.051	0.0000858	0.0000856	1.986	0.0004173	0.0013259
New Caledonia	158	152.731	0.0005490	0.0005307	0.129	0.0000000	0.0008453
New Zealand	7135	6536.841	0.0013909	0.0012743	1.248	0.0000000	0.0001909

Table S11: Raw and fitted case and death rates, on Tuesday, 21 June 2022, relative to cases 14 days earlier (Part 5).

location	cases	fitted cases	raw case rate	fitted case rate	fitted deaths	raw death rate	fitted death rate
Nicaragua	0	0.626	0.0000000	0.0000001	0.001	NaN	0.0018526
Niger	0	2.237	0.0000000	0.0000001	0.004	NaN	0.0018299
Nigeria	79	76.372	0.0000004	0.0000004	0.087	0.0000000	0.0011446
North Korea	0	2.300	0.0000000	0.0000001	0.004	NaN	0.0018290
North Macedonia	53	54.627	0.0000252	0.0000260	0.070	0.0000000	0.0012874
Norway	389	411.173	0.0000720	0.0000761	0.220	0.0000000	0.0005339
Oman	0	0.425	0.0000000	0.0000001	0.001	NaN	0.0018554
Pakistan	64	82.814	0.0000003	0.0000004	0.092	0.0000000	0.0011088
Palau	43	48.421	0.0023857	0.0026865	0.065	0.0000000	0.0013354
Palestine	174	158.193	0.0000339	0.0000308	0.132	NaN	0.0008315
Panama	3523	3134.744	0.0008096	0.0007204	4.486	0.0019231	0.0014309
Papua New Guinea	1	0.931	0.0000001	0.0000001	0.002	NaN	0.0018483
Paraguay	0	0.614	0.0000000	0.0000001	0.001	NaN	0.0018528
Peru	475	475.590	0.0000141	0.0000141	6.770	0.0046860	0.0142353
Philippines	168	186.963	0.0000015	0.0000016	2.663	0.0221130	0.0142417
Poland	338	330.305	0.0000088	0.0000086	4.682	0.0187166	0.0141744
Portugal	70701	70700.999	0.0068708	0.0068708	13.490	0.0012199	0.0001908
Qatar	316	305.981	0.0001175	0.0001138	0.187	0.0000000	0.0006125
Romania	404	425.725	0.0000209	0.0000220	0.224	NaN	0.0005250
Russia	3194	3196.351	0.0000220	0.0000220	45.526	0.0238480	0.0142432
Rwanda	18	22.104	0.0000013	0.0000016	0.035	0.0000000	0.0015833
Saint Helena	0	0.004	0.0000000	0.0000007	0.000	NaN	0.0018615
Saint Kitts and Nevis	2	1.789	0.0000420	0.0000376	0.003	NaN	0.0018362
Saint Lucia	28	26.523	0.0001559	0.0001476	0.041	0.0000000	0.0015360
Saint Pierre and Miquelon	14	14.225	0.0023797	0.0024179	0.024	NaN	0.0016741
Saint Vincent and the Grenadines	45	49.935	0.0004313	0.0004786	0.362	0.0256410	0.0072528
Samoa	0	0.036	0.0000000	0.0000002	0.000	0.0000000	0.0018610
San Marino	0	0.010	0.0000000	0.0000003	0.000	0.0000000	0.0018614
Sao Tome and Principe	0	0.036	0.0000000	0.0000002	0.000	NaN	0.0018610
Saudi Arabia	952	1090.929	0.0000265	0.0000303	1.753	0.0017498	0.0016065
Senegal	3	1.705	0.0000002	0.0000001	0.003	0.0000000	0.0018373
Serbia	281	280.103	0.0000409	0.0000408	0.648	0.0019724	0.0023138
Seychelles	0	0.021	0.0000000	0.0000002	0.000	NaN	0.0018612
Sierra Leone	0	0.762	0.0000000	0.0000001	0.001	NaN	0.0018507
Singapore	4477	3928.888	0.0008209	0.0007204	0.763	0.0000000	0.0001942
Sint Maarten (Dutch part)	0	0.012	0.0000000	0.0000003	0.000	NaN	0.0018613
Slovakia	260	256.223	0.0000477	0.0000470	0.170	0.0000000	0.0006643

Table S12: Raw and fitted case and death rates, on Tuesday, 21 June 2022, relative to cases 14 days earlier (Part 6).

location	cases	fitted cases	raw case rate	fitted case rate	fitted deaths	raw death rate	fitted death rate
Slovenia	462	541.623	0.0002180	0.0002556	0.252	0.0000000	0.0004659
Solomon Islands	0	0.087	0.0000000	0.0000001	0.000	NaN	0.0018603
Somalia	0	1.514	0.0000000	0.0000001	0.003	NaN	0.0018400
South Africa	2788	2802.999	0.0000469	0.0000472	12.387	Inf	0.0044194
South Korea	13320	13268.986	0.0002570	0.0002560	19.218	0.0012266	0.0014484
South Sudan	0	0.963	0.0000000	0.0000001	0.002	NaN	0.0018478
Spain	33293	34210.588	0.0007011	0.0007204	145.819	0.0024286	0.0042624
Sri Lanka	11	7.787	0.0000005	0.0000004	0.014	0.0000000	0.0017550
Sudan	17	16.338	0.0000004	0.0000004	0.027	0.0000000	0.0016489
Suriname	126	116.051	0.0002056	0.0001893	1.617	0.0784314	0.0139295
Sweden	0	0.938	0.0000000	0.0000001	0.002	NaN	0.0018482
Switzerland	10295	11075.508	0.0011845	0.0012743	2.113	0.0000000	0.0001908
Syria	1	1.893	0.0000000	0.0000001	0.003	0.0000000	0.0018347
Taiwan	82990	82988.980	0.0034782	0.0034782	120.945	0.0020402	0.0014574
Tajikistan	0	0.876	0.0000000	0.0000001	0.002	NaN	0.0018490
Tanzania	0	5.631	0.0000000	0.0000001	0.010	NaN	0.0017835
Thailand	2224	2173.793	0.0000311	0.0000304	30.303	0.0122520	0.0139403
Timor	1	0.351	0.0000008	0.0000003	0.001	0.0000000	0.0018565
Togo	11	14.153	0.0000013	0.0000016	0.024	NaN	0.0016750
Tokelau	0	0.002	0.0000000	0.0000013	0.000	NaN	0.0018615
Tonga	121	119.057	0.0011413	0.0011230	0.113	NaN	0.0009486
Trinidad and Tobago	233	222.064	0.0001527	0.0001456	2.548	0.0206897	0.0114723
Tunisia	668	733.700	0.0000545	0.0000598	10.450	0.0065876	0.0142429
Turkey	0	7.507	0.0000000	0.0000001	0.013	NaN	0.0017587
Turks and Caicos Islands	0	0.012	0.0000000	0.0000003	0.000	NaN	0.0018613
Uganda	34	16.438	0.0000007	0.0000004	0.227	0.0714286	0.0138298
Ukraine	0	3.855	0.0000000	0.0000001	0.035	0.0384615	0.0089671
United Arab Emirates	572	571.521	0.0000611	0.0000610	0.259	0.0000000	0.0004531
United Kingdom	10237	9526.019	0.0001522	0.0001416	135.681	0.0063907	0.0142432
United States	86732	86274.452	0.0002574	0.0002560	367.736	0.0040366	0.0042624
Uruguay	9184	9177.532	0.0026805	0.0026786	1.751	NaN	0.0001908
Uzbekistan	6	3.294	0.0000002	0.0000001	0.006	0.0000000	0.0018153
Vanuatu	178	169.646	0.0005578	0.0005316	0.137	0.0000000	0.0008047
Vatican	0	0.001	0.0000000	0.0000021	0.000	NaN	0.0018615
Venezuela	32	46.297	0.0000011	0.0000016	0.342	0.0078740	0.0073879
Vietnam	960	954.428	0.0000098	0.0000098	0.326	0.0000000	0.0003414
Wallis and Futuna	0	0.006	0.0000000	0.0000005	0.000	NaN	0.0018614
Yemen	0	2.921	0.0000000	0.0000001	0.005	0.0000000	0.0018204
Zambia	355	332.397	0.0000182	0.0000171	0.665	0.0046729	0.0020006
Zimbabwe	129	137.188	0.0000081	0.0000086	1.365	0.0227273	0.0099501

Table S13: Matrix V for data from Tuesday, 21 June 2022 using the 14-day lag, along with MAP classifications (Part 1)

	1	2	3	4	$\hat{\ell}$
Afghanistan	0.020	0.174	0.230	0.577	4
Albania	0.313	0.369	0.172	0.145	2
Algeria	0.259	0.324	0.174	0.243	2
Andorra	0.254	0.320	0.174	0.252	2
Angola	0.258	0.323	0.174	0.245	2
Anguilla	0.254	0.320	0.174	0.252	2
Antigua and Barbuda	0.254	0.320	0.174	0.252	2
Argentina	0.259	0.324	0.174	0.243	2
Armenia	0.254	0.320	0.174	0.251	2
Aruba	0.254	0.320	0.174	0.252	2
Australia	0.000	1.000	0.000	0.000	2
Austria	0.000	0.839	0.161	0.000	2
Azerbaijan	0.255	0.321	0.174	0.250	2
Bahamas	0.277	0.340	0.175	0.208	2
Bahrain	0.746	0.247	0.007	0.000	1
Bangladesh	0.000	0.025	0.099	0.875	4
Barbados	0.351	0.392	0.164	0.093	2
Belarus	0.255	0.321	0.174	0.250	2
Belgium	1.000	0.000	0.000	0.000	1
Belize	0.054	0.397	0.348	0.201	2
Benin	0.255	0.321	0.174	0.249	2
Bermuda	0.254	0.320	0.174	0.252	2
Bhutan	0.254	0.320	0.174	0.252	2
Bolivia	0.502	0.409	0.085	0.004	1
Bonaire Sint Eustatius and Saba	0.254	0.320	0.174	0.252	2
Bosnia and Herzegovina	0.286	0.348	0.175	0.191	2
Botswana	0.000	0.058	0.942	0.000	3
Brazil	0.000	0.000	1.000	0.000	3
British Virgin Islands	0.254	0.320	0.174	0.252	2
Brunei	0.508	0.407	0.082	0.003	1
Bulgaria	0.000	0.009	0.079	0.912	4
Burkina Faso	0.256	0.322	0.174	0.247	2
Burundi	0.255	0.321	0.174	0.249	2
Cambodia	0.256	0.322	0.174	0.248	2
Cameroon	0.257	0.323	0.174	0.246	2
Canada	0.000	0.126	0.874	0.000	3

Table S14: Matrix V for data from Tuesday, 21 June 2022 using the 14-day lag, along with MAP classifications (Part 2)

	1	2	3	4	$\hat{\ell}$
Cape Verde	0.302	0.360	0.174	0.164	2
Cayman Islands	0.254	0.320	0.174	0.252	2
Central African Republic	0.255	0.321	0.174	0.251	2
Chad	0.256	0.322	0.174	0.248	2
Chile	0.000	0.993	0.007	0.000	2
China	0.377	0.405	0.154	0.063	2
Colombia	0.259	0.325	0.174	0.241	2
Comoros	0.254	0.320	0.174	0.252	2
Congo	0.255	0.321	0.174	0.251	2
Cook Islands	0.269	0.334	0.175	0.222	2
Costa Rica	0.255	0.321	0.174	0.251	2
Cote d'Ivoire	0.257	0.323	0.174	0.246	2
Croatia	0.000	0.002	0.036	0.962	4
Cuba	0.275	0.339	0.175	0.211	2
Curacao	0.254	0.320	0.174	0.252	2
Cyprus	0.254	0.320	0.174	0.252	2
Czechia	0.490	0.413	0.092	0.005	1
Democratic Republic of Congo	0.264	0.329	0.175	0.232	2
Denmark	0.019	0.926	0.054	0.000	2
Djibouti	0.254	0.320	0.174	0.252	2
Dominica	0.254	0.320	0.174	0.252	2
Dominican Republic	0.853	0.146	0.001	0.000	1
Ecuador	0.000	0.013	0.058	0.929	4
Egypt	0.265	0.330	0.175	0.230	2
El Salvador	0.096	0.567	0.306	0.032	2
Equatorial Guinea	0.257	0.323	0.174	0.245	2
Eritrea	0.254	0.320	0.174	0.251	2
Estonia	0.020	0.595	0.380	0.005	2
Eswatini	0.304	0.362	0.173	0.161	2
Ethiopia	0.110	0.602	0.273	0.015	2
Faeroe Islands	0.254	0.320	0.174	0.252	2
Falkland Islands	0.254	0.320	0.174	0.252	2
Fiji	0.254	0.320	0.174	0.252	2
Finland	0.255	0.321	0.174	0.251	2
France	0.000	0.000	1.000	0.000	3
French Polynesia	0.254	0.320	0.174	0.252	2

Table S15: Matrix V for data from Tuesday, 21 June 2022 using the 14-day lag, along with MAP classifications (Part 3)

	1	2	3	4	$\hat{\ell}$
Gabon	0.254	0.320	0.174	0.251	2
Gambia	0.254	0.320	0.174	0.251	2
Georgia	0.581	0.373	0.046	0.000	1
Germany	0.000	1.000	0.000	0.000	2
Ghana	0.313	0.369	0.172	0.146	2
Gibraltar	0.254	0.320	0.174	0.252	2
Greece	0.000	0.280	0.720	0.000	3
Greenland	0.254	0.320	0.174	0.252	2
Grenada	0.023	0.199	0.250	0.528	4
Guatemala	0.751	0.243	0.006	0.000	1
Guernsey	0.254	0.320	0.174	0.252	2
Guinea	0.255	0.321	0.174	0.249	2
Guinea-Bissau	0.261	0.326	0.174	0.239	2
Guyana	0.376	0.404	0.155	0.065	2
Haiti	0.301	0.360	0.174	0.165	2
Honduras	0.255	0.321	0.174	0.250	2
Hong Kong	0.443	0.419	0.120	0.018	1
Hungary	0.255	0.321	0.174	0.250	2
Iceland	0.516	0.405	0.077	0.003	1
India	0.000	0.794	0.206	0.000	2
Indonesia	0.000	0.004	0.128	0.868	4
Iran	0.000	0.001	0.023	0.976	4
Iraq	0.403	0.414	0.143	0.041	2
Ireland	0.255	0.321	0.174	0.251	2
Isle of Man	0.254	0.320	0.174	0.252	2
Israel	0.998	0.002	0.000	0.000	1
Italy	0.000	1.000	0.000	0.000	2
Jamaica	0.410	0.415	0.139	0.036	2
Japan	0.000	1.000	0.000	0.000	2
Jersey	0.254	0.320	0.174	0.252	2
Jordan	0.255	0.321	0.174	0.250	2
Kazakhstan	0.290	0.351	0.175	0.185	2
Kenya	0.399	0.412	0.145	0.044	2
Kiribati	0.277	0.341	0.175	0.207	2
Kosovo	0.254	0.320	0.174	0.252	2
Kuwait	0.562	0.384	0.054	0.001	1

Table S16: Matrix V for data from Tuesday, 21 June 2022 using the 14-day lag, along with MAP classifications (Part 4)

	1	2	3	4	$\hat{\ell}$
Kyrgyzstan	0.255	0.321	0.174	0.251	2
Laos	0.269	0.333	0.175	0.223	2
Latvia	0.000	0.000	0.014	0.986	4
Lebanon	0.000	0.000	0.006	0.994	4
Lesotho	0.254	0.320	0.174	0.251	2
Liberia	0.255	0.321	0.174	0.251	2
Libya	0.255	0.321	0.174	0.251	2
Liechtenstein	0.295	0.355	0.174	0.176	2
Lithuania	0.437	0.419	0.124	0.020	1
Luxembourg	0.000	0.012	0.058	0.930	4
Macao	0.254	0.320	0.174	0.252	2
Madagascar	0.257	0.323	0.174	0.246	2
Malawi	0.263	0.328	0.175	0.235	2
Malaysia	0.038	0.760	0.202	0.000	2
Maldives	0.254	0.320	0.174	0.252	2
Mali	0.257	0.322	0.174	0.247	2
Malta	0.000	0.000	0.004	0.996	4
Marshall Islands	0.254	0.320	0.174	0.252	2
Mauritania	0.263	0.328	0.175	0.234	2
Mauritius	0.010	0.096	0.153	0.740	4
Mexico	0.000	0.000	0.768	0.232	3
Micronesia (country)	0.254	0.320	0.174	0.252	2
Moldova	0.254	0.320	0.174	0.251	2
Monaco	0.299	0.358	0.174	0.169	2
Mongolia	0.254	0.320	0.174	0.251	2
Montenegro	0.333	0.382	0.169	0.117	2
Montserrat	0.254	0.320	0.174	0.252	2
Morocco	0.618	0.350	0.032	0.000	1
Mozambique	0.024	0.210	0.259	0.507	4
Myanmar	0.260	0.326	0.174	0.240	2
Namibia	0.254	0.320	0.174	0.251	2
Nauru	0.254	0.320	0.174	0.252	2
Nepal	0.260	0.325	0.174	0.240	2
Netherlands	0.078	0.862	0.060	0.000	2
New Caledonia	0.396	0.412	0.146	0.046	2
New Zealand	1.000	0.000	0.000	0.000	1

Table S17: Matrix V for data from Tuesday, 21 June 2022 using the 14-day lag, along with MAP classifications (Part 5)

	1	2	3	4	$\hat{\ell}$
Nicaragua	0.255	0.321	0.174	0.250	2
Niger	0.257	0.323	0.174	0.247	2
Nigeria	0.335	0.383	0.168	0.114	2
North Korea	0.257	0.323	0.174	0.247	2
North Macedonia	0.314	0.369	0.172	0.145	2
Norway	0.532	0.398	0.068	0.002	1
Oman	0.254	0.321	0.174	0.251	2
Pakistan	0.341	0.387	0.167	0.106	2
Palau	0.308	0.365	0.173	0.155	2
Palestine	0.400	0.413	0.144	0.043	2
Panama	0.012	0.982	0.006	0.000	2
Papua New Guinea	0.255	0.321	0.174	0.250	2
Paraguay	0.255	0.321	0.174	0.251	2
Peru	0.000	0.000	0.000	1.000	4
Philippines	0.000	0.000	0.000	1.000	4
Poland	0.000	0.000	0.004	0.996	4
Portugal	1.000	0.000	0.000	0.000	1
Qatar	0.484	0.414	0.095	0.007	1
Romania	0.538	0.396	0.065	0.001	1
Russia	0.000	0.000	0.000	1.000	4
Rwanda	0.279	0.342	0.175	0.203	2
Saint Helena	0.254	0.320	0.174	0.252	2
Saint Kitts and Nevis	0.256	0.322	0.174	0.248	2
Saint Lucia	0.284	0.347	0.175	0.194	2
Saint Pierre and Miquelon	0.271	0.335	0.175	0.220	2
Saint Vincent and the Grenadines	0.017	0.153	0.211	0.620	4
Samoa	0.254	0.320	0.174	0.252	2
San Marino	0.254	0.320	0.174	0.252	2
Sao Tome and Principe	0.254	0.320	0.174	0.252	2
Saudi Arabia	0.043	0.786	0.171	0.000	2
Senegal	0.256	0.322	0.174	0.248	2
Serbia	0.071	0.479	0.347	0.103	2
Seychelles	0.254	0.320	0.174	0.252	2
Sierra Leone	0.255	0.321	0.174	0.250	2
Singapore	0.991	0.009	0.000	0.000	1
Sint Maarten (Dutch part)	0.254	0.320	0.174	0.252	2
Slovakia	0.459	0.418	0.111	0.012	1

Table S18: Matrix V for data from Tuesday, 21 June 2022 using the 14-day lag, along with MAP classifications (Part 6)

	1	2	3	4	$\hat{\ell}$
Slovenia	0.585	0.371	0.044	0.000	1
Solomon Islands	0.254	0.320	0.174	0.252	2
Somalia	0.256	0.322	0.174	0.248	2
South Africa	0.000	0.000	0.970	0.030	3
South Korea	0.003	0.997	0.000	0.000	2
South Sudan	0.255	0.321	0.174	0.250	2
Spain	0.000	0.000	1.000	0.000	3
Sri Lanka	0.263	0.328	0.175	0.234	2
Sudan	0.273	0.337	0.175	0.215	2
Suriname	0.000	0.001	0.017	0.982	4
Sweden	0.255	0.321	0.174	0.250	2
Switzerland	1.000	0.000	0.000	0.000	1
Syria	0.256	0.322	0.174	0.248	2
Taiwan	0.000	1.000	0.000	0.000	2
Tajikistan	0.255	0.321	0.174	0.250	2
Tanzania	0.261	0.326	0.174	0.239	2
Thailand	0.000	0.000	0.018	0.982	4
Timor	0.254	0.320	0.174	0.251	2
Togo	0.270	0.335	0.175	0.220	2
Tokelau	0.254	0.320	0.174	0.252	2
Tonga	0.371	0.403	0.157	0.069	2
Trinidad and Tobago	0.000	0.020	0.142	0.838	4
Tunisia	0.000	0.000	0.000	1.000	4
Turkey	0.263	0.328	0.175	0.235	2
Turks and Caicos Islands	0.254	0.320	0.174	0.252	2
Uganda	0.000	0.002	0.021	0.977	4
Ukraine	0.010	0.100	0.157	0.732	4
United Arab Emirates	0.596	0.364	0.040	0.000	1
United Kingdom	0.000	0.000	0.000	1.000	4
United States	0.000	0.000	1.000	0.000	3
Uruguay	1.000	0.000	0.000	0.000	1
Uzbekistan	0.258	0.324	0.174	0.244	2
Vanuatu	0.408	0.415	0.140	0.037	2
Vatican	0.254	0.320	0.174	0.252	2
Venezuela	0.016	0.148	0.206	0.629	4
Vietnam	0.719	0.271	0.010	0.000	1
Wallis and Futuna	0.254	0.320	0.174	0.252	2
Yemen	0.257	0.323	0.174	0.245	2
Zambia	0.084	0.528	0.330	0.058	2
Zimbabwe	0.001	0.058	0.184	0.757	4
