# Supplementary material for: Using Random Effect Models to Produce Robust Estimates of Death Rates in COVID-19 Data
Source: Int J Environ Res Public Health. 2022 Nov 14;19(22):14960. doi: 10.3390/ijerph192214960 (PMC9690214; doi:10.3390/ijerph192214960)
Supplement: Supplementary file 1 [file ijerph-19-14960-s001.zip › supp-S2.pdf]

Using random effect models to produce robust estimates of death rates in Covid-19 data

Section S2 R Code

load current data

```
load("covid.data.RData")
```

(The file `covid.data.RData` only loads two files: The data set `all.covid.data` as downloaded on 21/08/2022 by the authors, and a data frame `sanma.results` required for Section 4.3 which accelerates the compilation of this markdown notebook.)

source functions

```
daily.covid.rates <- function(date, lag=0,
                  deaths=TRUE,
                  digits=c(4,7),
                  K=c(30,4),
                  source.data = "https://covid.ourworldindata.org/data/owid-covid-data.csv"){

 if (is.character(source.data)){
   all.covid.data<- read.csv(source.data)
 } else {
   all.covid.data<-source.data
 }
 select <- c("location",
            "date",
            "new_cases",
            "new_deaths",
            "population"
           )
 non.countries <- c("International", "Europe", "Africa", "Asia", "European Union",
                   "Low income",  "Lower middle income", "Oceania", "South America",
                   "North America", "Upper middle income", "High income", "World")


 ex.pop <- which(is.na(all.covid.data$population))
 all.covid.data <- all.covid.data[-ex.pop,]

 date <- as.Date(date)
 if (lag<0){lag <- -lag}
```

```
lagdate <- max(date-lag, min(as.Date(all.covid.data$date)))


day.data <- all.covid.data[
   all.covid.data$date==date
   &!all.covid.data$location%in%non.countries,
   select]

day.data[is.na(day.data)]<-0

if (lag==0 || !deaths){
     day.lag.data<- day.data
 } else {
    day.lag.data <- all.covid.data[
      all.covid.data$date==lagdate
      &!all.covid.data$location%in%non.countries,
       select]
    day.lag.data <- day.lag.data[match(day.data$location, day.lag.data$location),]
    day.lag.data[is.na(day.lag.data)]<-0
}

require(npmlreg)

predict.cases<- function(fit){
  population<- exp(fit$offset)
  pred<- population*exp(post(fit)$int)
  return(pred)
}

k1<-K[1]
k2<-K[2]

day.fit <- alldist(new_cases~1 , random=~1, offset=log(population),
                   k=k1, data=day.lag.data,  family=poisson, tol=0.4,
                   plot.opt=0, verbose=FALSE)

predict.day.cases <- predict.cases(day.fit)
predict.day.case.rate <- predict.day.cases/day.lag.data$population

covid.day.table <- data.frame(
     "location" =  day.lag.data$location,
     "population" =  day.lag.data$population,
     "cases"= day.lag.data$new_cases,
     "fitted cases"= round(predict.day.cases,digits=digits[1]),
     "raw case rate" = round(day.lag.data$new_cases/day.lag.data$population,digits=digits[2]),
     "fitted case rate"= round(predict.day.case.rate, digits=digits[2])
)

if (deaths){
  deaths.fit <- alldist(new_deaths~1 , random=~1, offset=log(predict.day.cases),
                      k=k2,  data=day.data, family=poisson, tol=0.5,
                      plot.opt=0, verbose=FALSE)
```

```
predict.day.deaths <- predict.cases(deaths.fit)
predict.day.death.rate <- predict.day.deaths/predict.day.cases

covid.day.table <- data.frame(
     "location" =  day.lag.data$location,
     "population" =  day.lag.data$population,
     "cases"= day.lag.data$new_cases,
     "fitted cases"= round(predict.day.cases,digits=digits[1]),
     "raw case rate" = round(day.lag.data$new_cases/day.lag.data$population,digits=digits[2]),
     "fitted case rate"= round(predict.day.case.rate, digits=digits[2]),
     "deaths"=  day.data$new_deaths,
     "fitted deaths"= round(predict.day.deaths, digits=digits[1]),
     "raw death rate"= round(day.data$new_deaths/day.data$new_cases, digits=digits[2]),
     "fitted death rate"= round(predict.day.death.rate, digits=digits[2])
)
}

return(list(
   "covid.rates.table"=covid.day.table,
   "fit.cases"=day.fit,
   "fit.deaths"=deaths.fit))
}
```

Section 4.1

```
system.time(
  rates.20220621 <- daily.covid.rates("2022-06-21",
                              lag=0,
                              K=c(30,4),
                              deaths=TRUE,
                              digits=c(3,7),
                              source.data=all.covid.data)
  )
```

```
## Loading required package: npmlreg

##    user  system elapsed
##    5.61    0.32    5.98
```

```
system.time(
rates.20220621.lag14 <- daily.covid.rates("2022-06-21",
                              lag=14,
                              K=c(30,4),
                              deaths=TRUE,
                              digits=c(3,7),
                              source.data=all.covid.data )
)
```

```
##    user  system elapsed
##    7.08    0.22    7.36
```

```
head(rates.20220621$covid.rates.table)
```

```
##         location population cases fitted.cases raw.case.rate fitted.case.rate
## 849  Afghanistan   40099462    83       91.838      2.10e-06         2.30e-06
## 2677     Albania    2854710   219      229.237      7.67e-05         8.03e-05
## 3585     Algeria   44177969     8        2.766      2.00e-07         1.00e-07
## 4487     Andorra      79034     0        0.013      0.00e+00         2.00e-07
## 5371      Angola   34503774     0        2.160      0.00e+00         1.00e-07
## 6247    Anguilla      15753     0        0.005      0.00e+00         3.00e-07
##      deaths fitted.deaths raw.death.rate fitted.death.rate
## 849       1         0.359      0.0120482         0.0039064
## 2677      0         0.251      0.0000000         0.0010938
## 3585      0         0.005      0.0000000         0.0019398
## 4487      0         0.000            NaN         0.0019829
## 5371      0         0.004            NaN         0.0019490
## 6247      0         0.000            NaN         0.0019830
```

```
head(rates.20220621.lag14$covid.rates.table)
```

```
##         location population cases fitted.cases raw.case.rate fitted.case.rate
## 835  Afghanistan   40099462    53       65.833      1.30e-06         1.60e-06
## 2663     Albania    2854710    53       54.045      1.86e-05         1.89e-05
## 3571     Algeria   44177969     4        3.914      1.00e-07         1.00e-07
## 4473     Andorra      79034     0        0.017      0.00e+00         2.00e-07
## 5357      Angola   34503774     0        3.056      0.00e+00         1.00e-07
## 6233    Anguilla      15753     0        0.007      0.00e+00         4.00e-07
##      deaths fitted.deaths raw.death.rate fitted.death.rate
## 835       1         0.439      0.0120482         0.0066711
## 2663      0         0.070      0.0000000         0.0012919
## 3571      0         0.007      0.0000000         0.0018069
## 4473      0         0.000            NaN         0.0018615
## 5357      0         0.006            NaN         0.0018187
## 6233      0         0.000            NaN         0.0018616
```

Table S2

(uncommment `format="latex"` for LaTeX versions)

```
kable2 <-
  knitr::kable(head(rates.20220621$covid.rates.table, n=20),
                    # format="latex",
                    row.names=FALSE)
```

Table S3

```
kable3 <-
  knitr::kable(head(rates.20220621.lag14$covid.rates.table[,-c(2,7)], n=20),
                    # format="latex",
                    row.names=FALSE)
```

Tables for supplementary material

```
full.kable2 <- knitr::kable(rates.20220621$covid.rates.table,
                    # format="latex",
                    row.names=FALSE)
full.kable3 <- knitr::kable(rates.20220621.lag14$covid.rates.table[,-c(2,7)],
                    # format="latex",
                    row.names=FALSE)
```

Section 4.2

Table S4

(bottom two rows)

```
rates.20220621.lag14$fit.deaths$mass.points
```

```
##     MASS1     MASS2     MASS3     MASS4
## -8.564236 -6.531124 -5.457908 -4.251420
```

```
rates.20220621.lag14$fit.deaths$masses
```

```
##     MASS1     MASS2     MASS3     MASS4
## 0.2542821 0.3198613 0.1737598 0.2520968
```

(main part of table)

```
V14 <-
  cbind(round(post(rates.20220621.lag14$fit.deaths)$prob,digits=3),
        post(rates.20220621.lag14$fit.deaths)$classif)
V14.names <- rates.20220621.lag14$covid.rates.table[,1]
rownames(V14) <- V14.names
kable14 <- knitr::kable(head(V14,n=20),
                      #format="latex"
                      )
kable14
```

	1	2	3	4	
Afghanistan	0.020	0.174	0.230	0.577	4
Albania	0.313	0.369	0.172	0.145	2
Algeria	0.259	0.324	0.174	0.243	2
Andorra	0.254	0.320	0.174	0.252	2
Angola	0.258	0.323	0.174	0.245	2
Anguilla	0.254	0.320	0.174	0.252	2
Antigua and Barbuda	0.254	0.320	0.174	0.252	2
Argentina	0.259	0.324	0.174	0.243	2
Armenia	0.254	0.320	0.174	0.251	2

	1	2	3	4	
Aruba	0.254	0.320	0.174	0.252	2
Australia	0.000	1.000	0.000	0.000	2
Austria	0.000	0.839	0.161	0.000	2
Azerbaijan	0.255	0.321	0.174	0.250	2
Bahamas	0.277	0.340	0.175	0.208	2
Bahrain	0.746	0.247	0.007	0.000	1
Bangladesh	0.000	0.025	0.099	0.875	4
Barbados	0.350	0.392	0.164	0.093	2
Belarus	0.255	0.321	0.174	0.250	2
Belgium	1.000	0.000	0.000	0.000	1
Belize	0.054	0.397	0.348	0.201	2

(for the full version in the supplementary material, replace `n=20` by `n=221`)

MAP estimates:

```
table(apply(V14[,1:4],1,which.max))
```

```
##
##   1   2   3   4
##  26 159   9  28
```

Section 4.3

```
lag <- 14
index <- 308:672            # identifying the year 2021
lagged.index <- 308:672-lag
```

```
all.sanma.data <- all.covid.data[all.covid.data$location=="San Marino",]
Dates2021 <- all.sanma.data$date[index]
```

Existence of rates

zero-lag:

```
sum(is.na(all.sanma.data$new_deaths[index]/all.sanma.data$new_cases[index]))
```

```
## [1] 151
```

```
sum(all.sanma.data$new_deaths[index]/all.sanma.data$new_cases[index]==0,na.rm=TRUE )
```

```
## [1] 180
```

```
sum(all.sanma.data$new_deaths[index]/all.sanma.data$new_cases[index]> 0,na.rm=TRUE )
```

```
## [1] 34
```

14-day-lag:

```
sum(is.na(all.sanma.data$new_deaths[index]/all.sanma.data$new_cases[lagged.index]))
```

```
## [1] 146
```

```
sum(all.sanma.data$new_deaths[index]/all.sanma.data$new_cases[lagged.index]==0,na.rm=TRUE )
```

```
## [1] 185
```

```
sum(all.sanma.data$new_deaths[index]/all.sanma.data$new_cases[lagged.index]> 0,na.rm=TRUE )
```

```
## [1] 34
```

Figure S1 top row

```
plot(all.sanma.data$new_cases[index], ylab="cases", xlab="day")
```

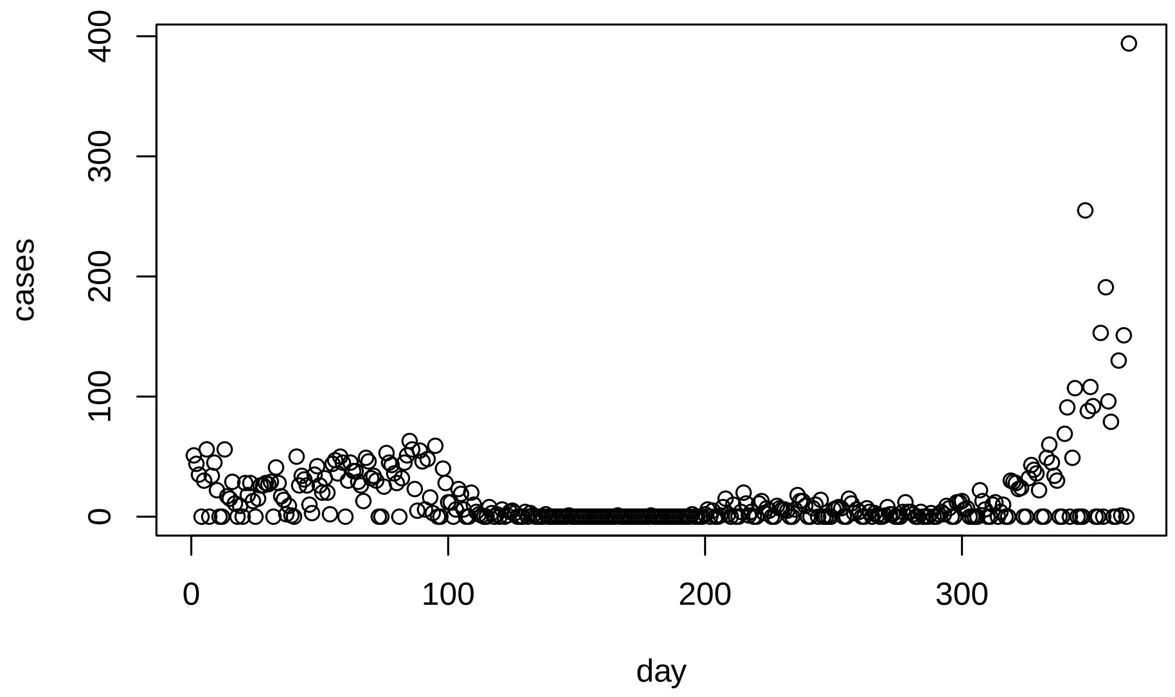


```
plot(all.sanma.data$new_deaths[index], ylab="deaths", xlab="day")
```

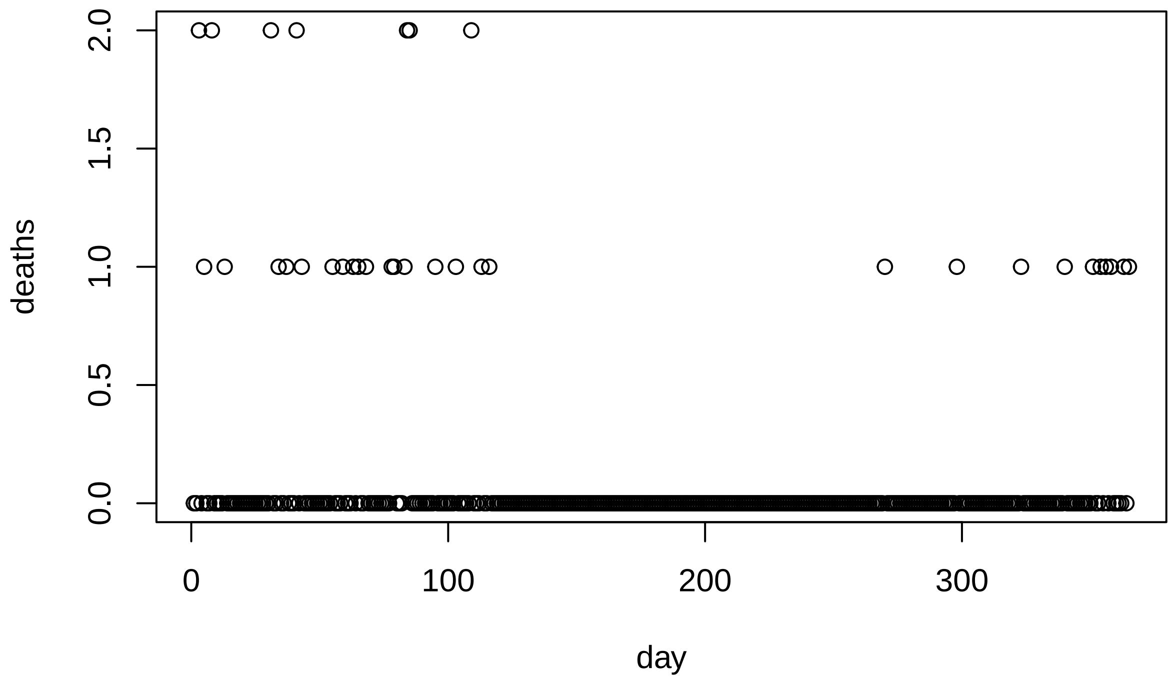

(The following are commented out since the matrix `sanma.results` has already been loaded.)

```
# sanma.results <- matrix(0,365,10)
# colnames(sanma.results)<- colnames(rates)
```

(The following only recalculates the first row of the matrix; it can be extended to all 365 countries by replacing `1:1` with `1:365`.)

```
 for  (j in 1:1){
   rates <- daily.covid.rates(Dates2021[j],
                              lag=0,
                              K=c(30,4),
                              deaths=TRUE,
                              digits=c(4,7),
                              source.data=all.covid.data)$covid.rates.table
  sanma.results[j,]<- unlist(rates[rates$location=="San Marino",])
print(j)
}
```

```
## [1] 1
```

Figure S1 middle row

```
plot(as.numeric(sanma.results[,"fitted.case.rate"]), ylab="fitted case rates", xlab="day")
```

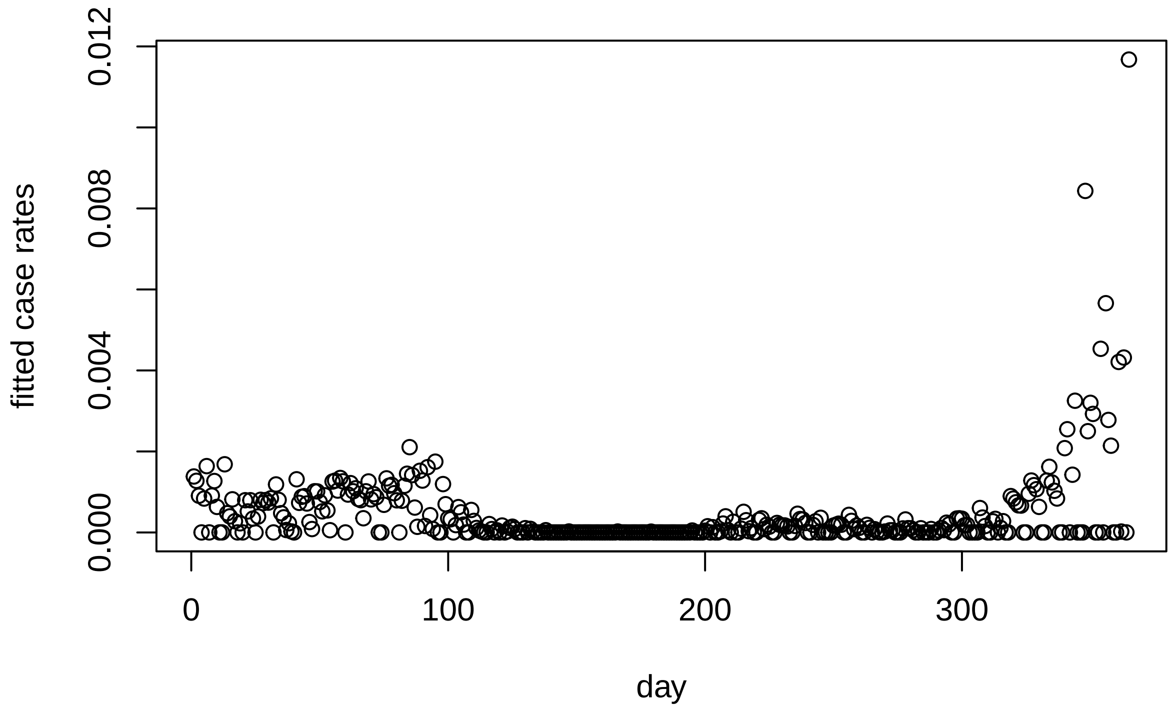


```
plot(as.numeric(sanma.results[,"fitted.death.rate"]), ylab="fitted death rates", xlab="day")
```

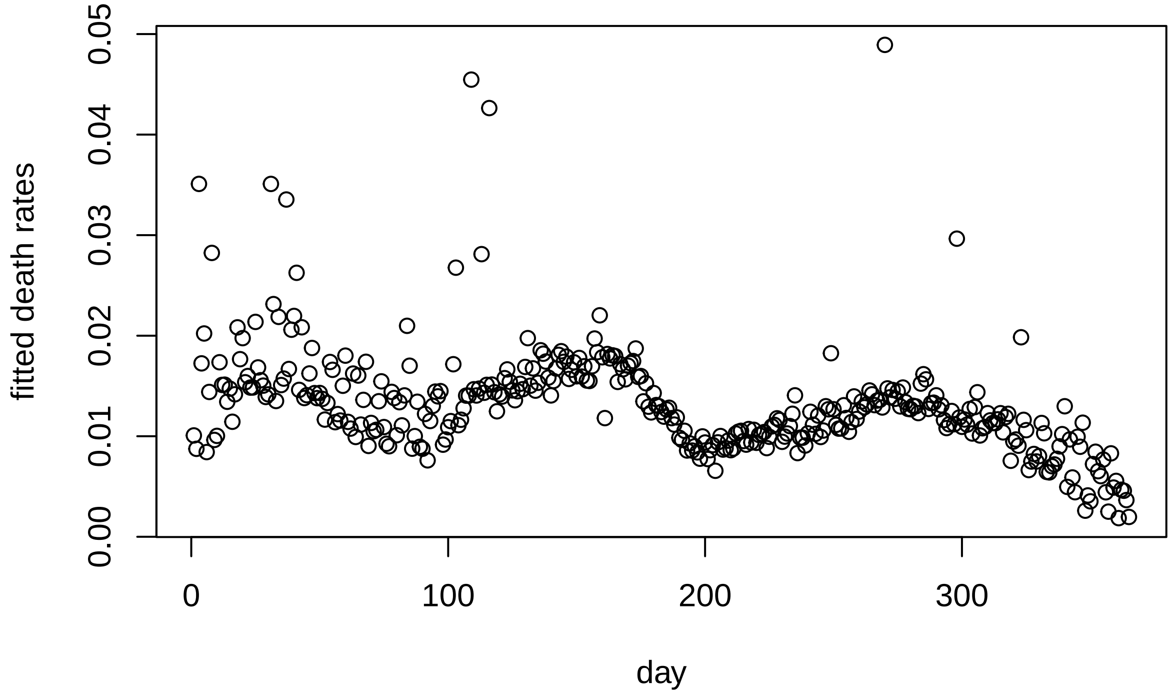

Figure S1 bottom row

```
plot(as.numeric(sanma.results[,"raw.case.rate"]),
     as.numeric(sanma.results[,"fitted.case.rate"]),
     ylab="fitted", xlab="raw", main="case rates")
abline(0,1, col=2)
```

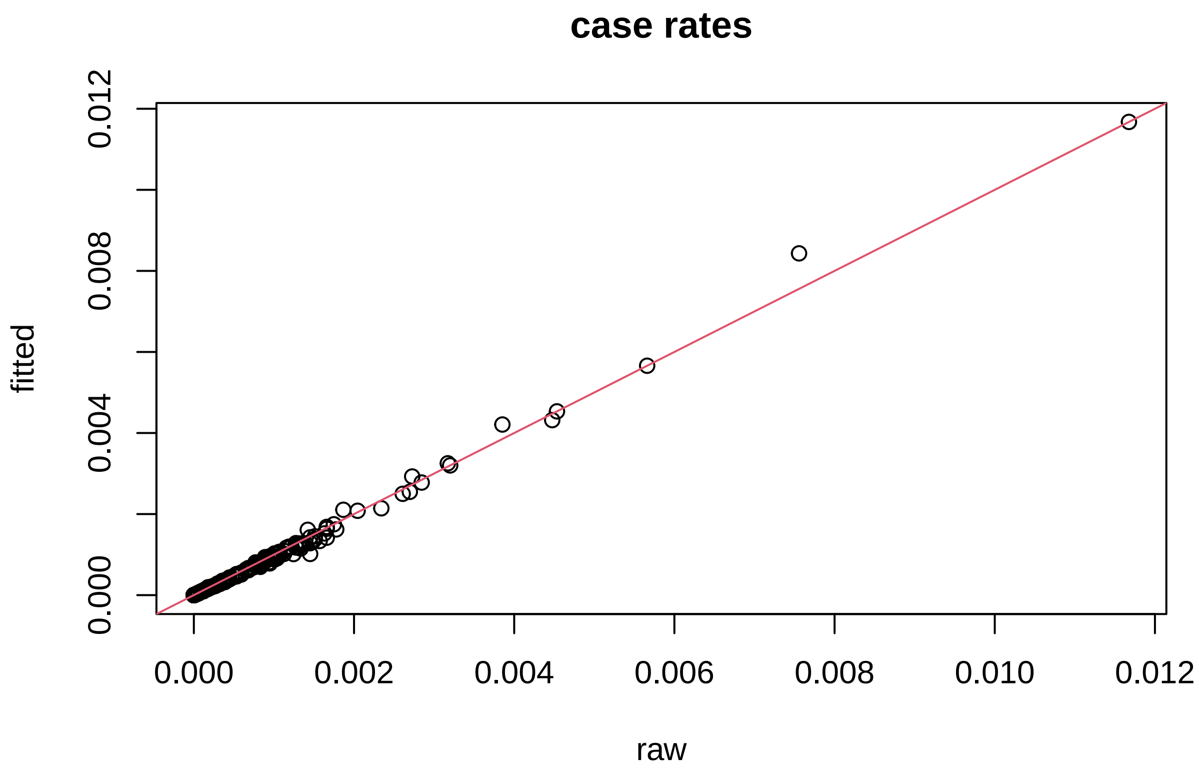


```
plot(as.numeric(sanma.results[,"raw.death.rate"]),
     as.numeric(sanma.results[,"fitted.death.rate"]),
     ylab="fitted", xlab="raw", main="death rates")
abline(0,1, col=2)
```

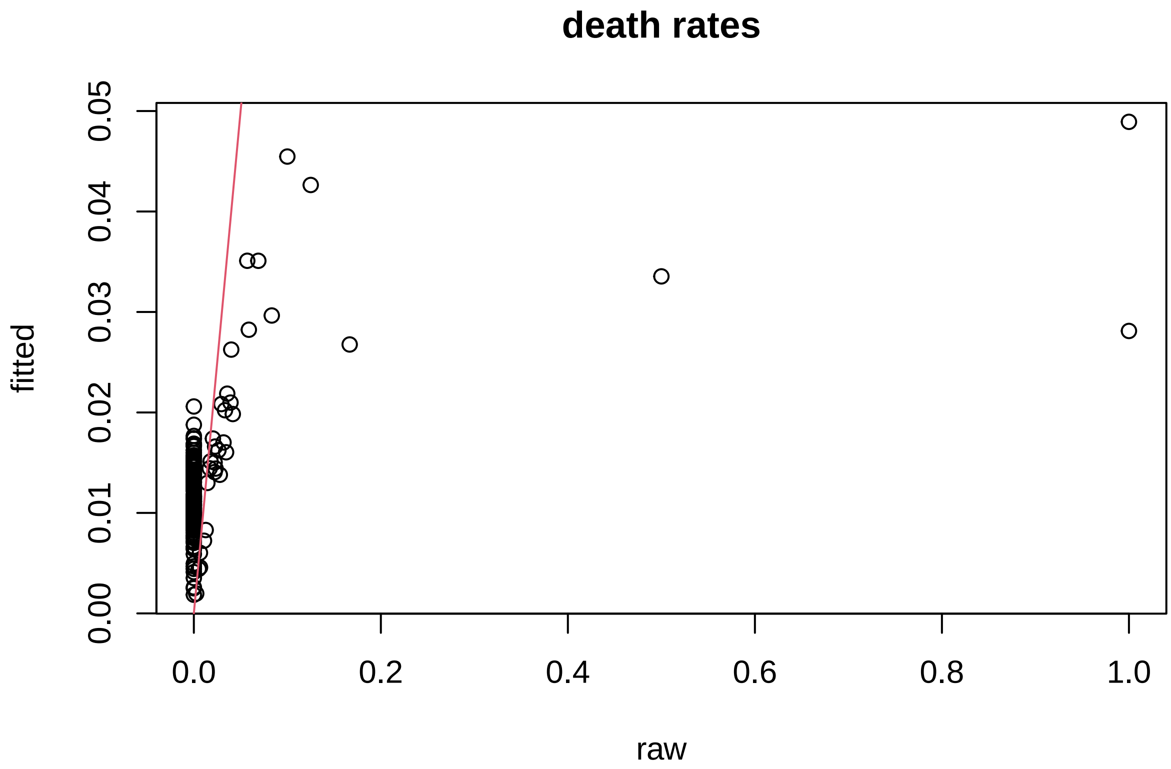

Section 4.4

Analysis is identical as for the San Marino data. Just use the following in the second chunk of Section 4.3.

```
# all.saudi.data <- all.covid.data[all.covid.data$location=="Saudi Arabia",]
```
